# Enrichment analysis applied to disease prognosis

**DOI:** 10.1186/2041-1480-4-21

**Published:** 2013-10-08

**Authors:** Catia M Machado, Ana T Freitas, Francisco M Couto

**Affiliations:** 1 LaSIGE, Departamento de Informática, Faculdade de Ciências, Universidade de Lisboa, Lisboa, Portugal; 2 Instituto de Engenharia de Sistemas e Computadores/Instituto Superior Técnico, Lisboa, Portugal

## Abstract

Enrichment analysis is well established in the field of transcriptomics, where it is used to identify relevant biological features that characterize a set of genes obtained in an experiment.

This article proposes the application of enrichment analysis as a first step in a disease prognosis methodology, in particular of diseases with a strong genetic component. With this analysis the objective is to identify clinical and biological features that characterize groups of patients with a common disease, and that can be used to distinguish between groups of patients associated with disease-related events. Data mining methodologies can then be used to exploit those features, and assist medical doctors in the evaluation of the patients in respect to their predisposition for a specific event.

In this work the disease hypertrophic cardiomyopathy (HCM) is used as a case-study, as a first test to assess the feasibility of the application of an enrichment analysis to disease prognosis. To perform this assessment, two groups of patients have been considered: patients that have suffered a sudden cardiac death episode and patients that have not.

The results presented were obtained with genetic data and the Gene Ontology, in two enrichment analyses: an enrichment profiling aiming at characterizing a group of patients (e.g. that suffered a disease-related event) based on their mutations; and a differential enrichment aiming at identifying differentiating features between a sub-group of patients and all the patients with the disease. These analyses correspond to an adaptation of the standard enrichment analysis, since multiple sets of genes are being considered, one for each patient.

The preliminary results are promising, as the sets of terms obtained reflect the current knowledge about the gene functions commonly altered in HCM patients, thus allowing their characterization. Nevertheless, some factors need to be taken into consideration before the full potential of the enrichment analysis in the prognosis methodology can be evaluated. One of such factors is the need to test the enrichment analysis with clinical data, in addition to genetic data, since both types of data are expected to be necessary for prognosis purposes.

## Background

Enrichment analysis is extensively used for the functional analysis of large lists of genes identified with high-throughput technologies, such as expression microarrays. It exploits the use of statistical methods over ontological gene annotations to identify biological features that are represented in a gene set under analysis more than would be expected by chance. Such biological features are said to be enriched, or overrepresented, in the study set and are then used to formulate a biological interpretation about it.

The ontology most commonly used in these analyses is the Gene Ontology [1-3], although other resources such as MeSH and KEGG have also been explored [4]. Strategies based on multiple vocabularies have also been developed, namely in pharmacogenomics considering the Human Disease Ontology and the Pharmacogenomics Knowledge Base [5]. LePendu et al. [6] proposed a method to generate annotations when using medical vocabularies, testing its feasibility with the Human Disease Ontology.

Enrichment analyses are normally divided in three categories: Singular Enrichment Analysis (SEA), Gene Set Enrichment Analysis (GSEA) and Modular Enrichment Analysis (MEA). SEA works with a user-selected gene set and iteratively tests the enrichment of each individual ontology concept in a linear mode. GSEA also evaluates the enrichment of ontology concepts individually, but considering all the genes in the experiment and not just a user-selected gene set. MEA works with a user-selected gene set, but incorporates into the analysis the relationships between concepts represented in the ontologies, thus evolving from a term-centric approach to a biological module-centric approach [7]. Several tools have been developed that implement one or more of these approaches, such as Ontologizer [8,9], Onto-express [10] and GSEA [11].

**Figure 1:**
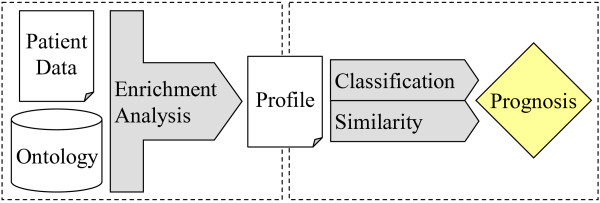
**Schematic representation of the prognosis methodology.** The methodology is composed by two units: the first (left-side) receives as input data from patients mapped to biomedical ontologies/controlled vocabularies. It performs an enrichment analysis to identify a list of ontology terms considered to be enriched, which will be used to create profiles for individual patients. These profiles will then be subjected to an evaluation step (the second unit, on the right-side) that will result in the evaluation of the prognosis for the patients. For the implementation of the second unit, both a classification and a similarity approach will be explored.

This article proposes the application of enrichment analysis for disease prognosis, as the first component of a prognosis methodology that will assist in the evaluation of patients in respect to the likelihood of suffering a disease-related event (see Figure 1). By performing an enrichment analysis on the patients’ data based on controlled vocabularies, we expect to identify sets of characterizing features that will be used as profiles for the patients. These profiles will then be explored to evaluate the predisposition of the patients for the specific event. This evaluation is the second component of the prognosis methodology, and can be performed by following a classification or a similarity approach. In the classification approach, the terms composing the profiles will be added as features to the patients’ dataset and analyzed with classification algorithms such as random forests [12] and Bayesian networks [13]. In the similarity approach, semantic similarity measures will be used to compute the similarity between patients, based on their profiles. Different semantic similarity measures [14] and a relatedness measure [15] can be explored to compare the patients’ profiles.

The purpose of the prognosis methodology is to assist medical doctors in the definition of the appropriate treatments and preventive actions for individual patients. Consequently, the datasets to be used with this methodology are collected by biomedical experts in the context of medical practice. Such datasets are frequently characterized by a small number of clinical features and a high number of missing values, which difficults their use for knowledge extraction purposes. We propose that the application of an enrichment analysis to this type of dataset can result in the extraction of relevant knowledge from controlled vocabularies to improve the quality of the dataset and, therefore, improve the quality of the predictions made with it.

The present work focuses in diseases which diagnosis and prognosis are dependent both on clinical and genetic data. An example of such a disease is hypertrophic cardiomyopathy (HCM), which is used in this work as a case-study. HCM is an autosomal dominant genetic disease, characterized by a variable clinical presentation and onset, with approximately 900 mutations in more than 30 genes currently known to be associated with it [16]. It has been observed that the presence of a given mutation can correspond to a benign manifestation in one individual and can result in sudden cardiac death (SCD) in another [17,18]. This disease is, in fact, the most frequent cause of SCD in apparently healthy young people and athletes [17,18]. Given the severity of this manifestation of the disease, SCD is the event evaluated with our prognosis methodology.

This methodology is under development, and in the present article we present the preliminary results obtained when applying the enrichment analysis to the genetic data of patients with HCM. While standard applications of enrichment analysis analyze a single set of genes per experiment, the application here proposed analyzes several sets of genes, one from each patient. Our implementation of the enrichment analysis had thus to be adapted to accommodate multiple sets of genes.

The Gene Ontology (GO) was the ontology chosen to perform the analysis instead of other genetic vocabularies since it is the most well studied application of enrichment analysis. It allows the annotation of biological products with terms describing the molecular functions they perform, the biological processes in which they are involved, and the cellular components where they are located or of which they are a component. Additionally, the GO was chosen instead of a clinical vocabulary since it has been extensively used for annotation purposes, and thus possesses a background set of annotations that can be promptly used, which is not normally the case for clinical vocabularies.

The following sections present and discuss the results obtained with the adapted enrichment analysis, considering the genetic data of the HCM patients and the GO; delineate the conclusions extracted from the results, as well as how the work will evolve; and explain in detail the methods followed to obtain the results presented.

## Results and discussion

Since all the patients share the same genome, it is through their individual mutations that we can find differentiating features. However, information regarding a patient’s mutations, when available, exists only for a few genes. In the case of the HCM patients, the genetic data used in this analysis is precisely the presence/absence of the mutations in the genes associated with the disease.

An oversimplified way to define the study set when analyzing, for example, the SCD patients, would be to consider the list of genes mutated in at least one of these patients. However, this would only be accurate if all the SCD patients had a mutation in those genes, which might not be the truth. In order to maximize our use of the available genetic information, the best option is to consider the set of mutations each patient has, individually.

Following this approach, we performed two enrichment analyses: a profiling analysis, where the total number of genes mutated in each group of patients (with SCD and without SCD) was compared with all (protein-coding) genes in the same group of patients; and a differential analysis, where the total number of genes mutated in each group of patients (with SCD and without SCD) was compared with the total number of genes mutated in all the HCM patients.

### Enrichment profiling

Terms identified as enriched by the profiling analysis can be used to characterize the genotype of patients with and without SCD (SCD and no-SCD, respectively), since they correspond to specific functional aspects that are mutated in the patients. These functional aspects, in turn, correspond to phenotypical traits expected to be altered. While terms identified as enriched both in SCD and no-SCD patients can be interpreted as associated with the disease, terms enriched differently can be interpreted as associated with the occurrence of SCD. This profiling analysis is more directly comparable with the application of enrichment analysis to gene expression data, where a set of genes (e.g. overexpressed) can be analyzed against the whole genome.

**Figure 2:**
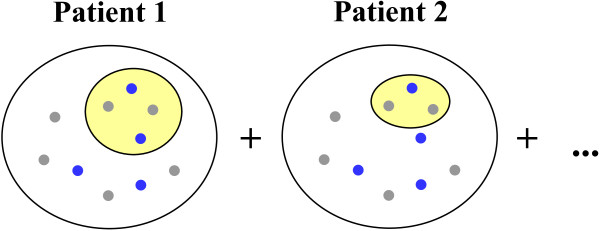
**Representation of the population and study sets in the enrichment profiling analysis.** The two sets of dots represent the genome of two patients, from the same group (e.g. with SCD). The smaller, yellow set of dots, corresponds to the genes mutated in the patient; the larger, white set of dots, corresponds to the entire genome of the patient: genes not mutated (outside the yellow set) and genes mutated. In these sets of genes, blue dots represent genes annotated with a term of interest (*t*); gray dots represent genes not annotated with *t*. In the profiling analysis, the study set is the union of the genes mutated in all the patients; the population set is the union of the genome of all the patients. The annotation frequency is then calculated by counting the total number of genes annotated with the term in the study set (study frequency) and in the population set (population frequency).

In the enrichment approach followed in this work, i.e. Single Enrichment Analysis, the set of genes selected by the user to be evaluated for the existence of enriched ontology terms is called the *study set*, and these genes can be the ones overexpressed in a microarray. The reference set of genes is called the *population set*, and can be the whole set of genes analyzed in the microarray. In the context of the patients’ profiling analysis, we can theorize the existence of a study set and a population set for each individual patient. The study set contains the genes mutated in the patient, whereas the population set contains all genes in the patient, either mutated or not. In the HCM dataset we only have mutation information for the genes associated with the disease, and consequently the study set is exclusively composed by these genes. The genes associated with HCM but not tested (see the Methods section for an explanation of how the genotyping is performed) have to be treated as genes without mutations just as happens with the genes not associated with HCM, and are included in the population set. The enrichment analysis is then performed considering in the study set all the genes mutated in all the patients of a given group (e.g. with SCD). In turn, the population set includes all the genes in the genome of all the patients in the same group (see Figure 2 for a representation of how the two sets of genes are obtained).

For SCD patients, the study set contains 16 mutated genes (total for the 14 SCD patients) and the population set contains 18,759 × 14 genes (the number of GO annotated protein-coding Human genes multiplied by the number of SCD patients). For no-SCD patients, the study set contains 100 mutated genes (total for the 69 no-SCD patients) and the population set contains 18,759 × 69 genes (see Table 1 for a compilation of the number of genes analyzed in both enrichment analyses). It is important to note that the SCD patients have mutations in only 4 distinct genes, and the no-SCD in 7 distinct genes that include the previous 4.

**Table 1:** Number of genes considered in the profiling and the differential enrichment analyses

**Enrichment test**		**Study set**	**Population set**
**Enrichment profiling**	**SCD**	16	18,759 × 14
	**no-SCD**	100	18,759 × 69
**Differential enrichment**	**SCD**	16	116
	**no-SCD**	100	116

For each enrichment test performed is indicated the number of genes in the study and the population sets.

As shown in Table 2 (in the column *Total*), the enrichment profiling analysis identified the following number of enriched terms (*p*-value <0.1): 53 for SCD and 70 for no-SCD, without multiple-testing correction; 40 for SCD and 62 for no-SCD, with Bonferroni correction.

**Table 2:** Number of enriched terms in each of the analyses performed

		**Number of enriched terms**							
**Analysis**		**Bio. Proc.**		**Mol. Func.**		**Cel. Comp.**		**Total**	
		**noCorr**	**Bonf**	**noCorr**	**Bonf**	**noCorr**	**Bonf**	**noCorr**	**Bonf**
**Profiling**	**SCD**	30	19	13	11	10	10	53	40
	**no-SCD**	39	33	21	19	10	10	70	62
**Differential**	**SCD vs no-SCD**	0	0	1	0	0	0	1	0
	**no-SCD vs SCD**	2	0	1	0	2	0	5	0

For each enrichment analysis is indicated the number of terms of each GO type (biological process, molecular function and cellular component), with *p*-value below 0.1, when considering no multiple-testing correction (noCorr) and with Bonferroni correction (Bonf).

Tables 3, 4 and 5 show, respectively, the top 10 enriched biological process (BP), molecular function (MF) and cellular component (CC) terms for the SCD patients. The top 10 enriched terms identified for the no-SCD patients are not shown since they are nearly identical to those of SCD. The full set of results for SCD and no-SCD is available in the Additional files 1 and 2, respectively.

**Table 3:** Top 10 enriched biological process terms in the profiling analysis of SCD patients

**Acc**	**Name**	***p*-value**	***p*-Bonf**	**SFreq**	**PFreq**
GO:0030049	Muscle filament sliding	7.7E-40	4.1E-38	94%	0.21%
GO:0033275	Actin-myosin filament sliding	7.7E-40	4.1E-38	94%	0.21%
GO:0055010	Ventricular cardiac muscle tissue morphogenesis	7.7E-40	4.1E-38	94%	0.21%
GO:0003229	Ventricular cardiac muscle tissue development	2.4E-39	1.3E-37	94%	0.22%
GO:0070252	Actin-mediated cell contraction	6.8E-39	3.6E-37	94%	0.24%
GO:0002027	Regulation of heart rate	1.3E-31	6.9E-30	81%	0.26%
GO:0007512	Adult heart development	6.8E-25	3.6E-23	56%	0.07%
GO:0032781	Positive regulation of ATPase activity	2.9E-15	1.5E-13	38%	0.09%
GO:0043462	Regulation of ATPase activity	2.6E-14	1.4E-12	38%	0.12%
GO:0032971	Regulation of muscle filament sliding	1.0E-12	5.4E-11	25%	0.02%

The terms shown are the 10 biological process terms with the lowest *p*-value, obtained in the profiling of SCD patients. For each term is indicated: GO accession number (Acc), term name, *p*-value without multiple-testing correction, *p*-value with Bonferroni correction (*p*-Bonf), annotation frequency in the study set (SFreq) and annotation frequency in the population set (PFreq).

**Table 4:** Top 10 enriched molecular function terms in the profiling analysis of SCD patients

**Acc**	**Name**	***p*-value**	***p*-Bonf**	**SFreq**	**PFreq**
GO:0008307	Structural constituent of muscle	2.9E-35	1.6E-33	88%	0.25%
GO:0030898	Actin-dependent ATPase activity	1.1E-26	6.1E-25	56%	0.05%
GO:0000146	Microfilament motor activity	1.8E-23	9.4E-22	56%	0.11%
GO:0032036	Myosin heavy chain binding	3.3E-11	1.8E-09	25%	0.04%
GO:0001671	ATPase activator activity	9.1E-11	4.8E-09	25%	0.05%
GO:0031432	Titin binding	2.1E-10	1.1E-08	25%	0.06%
GO:0060590	ATPase regulator activity	4.0E-10	2.1E-08	25%	0.07%
GO:0017022	Myosin binding	7.6E-09	4.0E-07	25%	0.14%
GO:0030172	Troponin C binding	5.3E-06	2.8E-04	13%	0.02%
GO:0031013	Troponin I binding	8.4E-06	4.4E-04	13%	0.03%

The terms shown are the 10 molecular function terms with the lowest *p*-value, obtained in the profiling of SCD patients. For each term is indicated: GO accession number (Acc), term name, *p*-value without multiple-testing correction, *p*-value with Bonferroni correction (*p*-Bonf), annotation frequency in the study set (SFreq) and annotation frequency in the population set (PFreq).

**Table 5:** Top 10 enriched cellular component terms in the profiling analysis of SCD patients

**Acc**	**Name**	***p*-value**	***p*-Bonf**	**SFreq**	**PFreq**
GO:0005859	Muscle myosin complex	2.4E-37	1.3E-35	81%	0.10%
GO:0032982	Myosin filament	2.4E-37	1.3E-35	81%	0.10%
GO:0016460	Myosin II complex	1.1E-35	5.8E-34	81%	0.13%
GO:0001725	Stress fiber	4.1E-20	2.2E-18	56%	0.25%
GO:0032432	Actin filament bundle	7.3E-20	3.8E-18	56%	0.27%
GO:0014705	C zone	9.2E-15	4.9E-13	25%	0.01%
GO:0005863	Striated muscle myosin thick filament	1.0E-12	5.4E-11	25%	0.02%
GO:0031672	A band	4.7E-09	2.5E-07	25%	0.13%
GO:0005861	Troponin complex	2.2E-05	1.1E-03	13%	0.04%
GO:0005865	Striated muscle thin filament	6.6E-05	3.5E-03	13%	0.07%

The terms shown are the 10 cellular component terms with the lowest *p*-value, obtained in the profiling of SCD patients. For each term is indicated: GO accession number (Acc), term name, *p*-value without multiple-testing correction, *p*-value with Bonferroni correction (*p*-Bonf), annotation frequency in the study set (SFreq) and annotation frequency in the population set (PFreq).

Analyzing the enriched terms in detail, we can confirm their relation with HCM. According to the BP terms enriched, the patients analyzed suffer from cardiac alterations (e.g. *regulation of heart rate*, *adult heart development*), in particular in the ventricle (*ventricular cardiac muscle tissue morphogenesis* and *ventricular cardiac muscle tissue development*), and some of those alterations affect the contraction of striated muscles, in which group the cardiac muscle is included (e.g. *actin-myosin filament sliding* and *actin-mediated cell contraction*). HCM is indeed a cardiac disease, in which the main anatomical manifestation is the thickening of the interventricular septum, and the occurrence of a sudden cardiac arrest can be a consequence of the malfunctioning of the heart contraction. Considering the MF terms, several *binding* terms are enriched, namely *myosin heavy chain binding*, *titin binding*, *troponin C* and *troponin I binding*. All of these terms refer to proteins that participate in the contraction of the filaments that compose striated muscles, and thus the HCM patients present alterations in the normal function of this type of muscle. Finally, the CC terms confirm the previous observations that the alterations in HCM patients occur at the level of striated muscle functioning, namely through the following terms: *striated muscle myosin thick filament*, *striated muscle thin filament*, *troponin complex*, *A band* (a component of the sarcomere) and *C zone* (a component of the A band).

The difference between terms enriched for SCD and for no-SCD consists in a set of 18 terms identified in the latter and not in the former (see Table 6). These terms do not provide biologically meaningful information, since it cannot be interpreted that when those functions and processes are altered the patients will not suffer a SCD episode. These differences in the set of enriched terms can be explained by the fact that the number of no-SCD patients is considerably larger than the number of SCD patients (69 vs. 14) and consequently there are more distinct genes mutated (7 vs. 4). Thus, the enrichment profiling analysis did not identify differentiating aspects between the two groups that can be used to prognosticate SCD solely based on genetic data.

**Table 6:** Enriched terms in the profiling analysis of no-SCD patients, not identified in the SCD patients

**Acc**	**Name**	***p*-value**	***p*-Bonf**	**SFreq**	**PFreq**	
	**Biological process**					
GO:0001980	Regulation of systemic arterial blood	6.0E-41	4.4E-39	13%	0.00	
	pressure by ischemic conditions					
GO:0001976	Neurological system process involved in regulation	3.4E-25	2.5E-23	13%	0.00	
	of systemic arterial blood pressure					
GO:0006940	Regulation of smooth muscle contraction	6.6E-19	4.9E-17	13%	0.00	
GO:0007522	Visceral muscle development	5.3E-03	3.9E-01	1%	0.00	
GO:0042694	Muscle cell fate specification	5.3E-03	3.9E-01	1%	0.00	
GO:0055009	Atrial cardiac muscle tissue morphogenesis	2.6E-02	1.9E+00	1%	0.00	
GO:0003228	Atrial cardiac muscle tissue development	2.6E-02	1.9E+00	1%	0.00	
GO:0048739	Cardiac muscle fiber development	4.2E-02	3.1E+00	1%	0.00	
GO:0042693	Muscle cell fate commitment	5.7E-02	4.2E+00	1%	0.00	
	**Molecular function**					
GO:0031014	Troponin T binding	9.9E-33	7.3E-31	13%	0.00	
GO:0019855	Calcium channel inhibitor activity	1.9E-31	1.4E-29	13%	0.00	
GO:0008200	Ion channel inhibitor activity	1.4E-23	1.0E-21	13%	0.00	
GO:0016248	Channel inhibitor activity	1.4E-23	1.0E-21	13%	0.00	
GO:0005246	Calcium channel regulator activity	4.9E-23	3.6E-21	13%	0.00	
GO:0048306	Calcium-dependent protein binding	1.1E-19	8.1E-18	13%	0.00	
GO:0042805	Actinin binding	5.6E-06	4.2E-04	4%	0.00	
GO:0030899	Calcium-dependent ATPase activity	1.6E-02	1	1%	0.00	
GO:0003785	Actin monomer binding	7.2E-02	1	1%	0.00	

The terms shown are the biological process and molecular function terms identified as enriched in the profiling analysis of no-SCD patients that were not identified in the profiling of SCD patients. For each term is indicated: GO accession number (Acc), term name, *p*-value without multiple-testing correction, *p*-value with Bonferroni correction (*p*-Bonf), annotation frequency in the study set (SFreq) and annotation frequency in the population set (PFreq).

### Differential enrichment

With the differential enrichment analysis, our purpose was to identify the differences between SCD and no-SCD, and thus compare each, in turn, with the complete set of HCM patients. Since this set is divided in SCD and no-SCD patients, we are basically comparing one group with the other. As happened in the enrichment profiling, the study set contains 16 and 100 genes mutated for SCD and no-SCD, respectively. As for the population set, in this case it contains 116 mutated genes (the sum of the genes mutated in SCD and in no-SCD). Given the design of the analysis, terms found enriched correspond to functional aspects that are mutated more frequently in one group of patients than in the other.

A total of one term for SCD and five terms for no-SCD were identified as enriched (*p*-value <0.1, not considering multiple-testing correction). The SCD term is the MF *structural constituent of muscle* (*p*-value = 0.08). The no-SCD terms are: *negative regulation of ATPase activity* (*p*-value = 0.08) and *regulation of ATPase activity* (*p*-value = 0.09; both BP); *striated muscle thin filament* (*p*-value = 0.08) and *troponin complex* (*p*-value = 0.08; both CC); and *troponin C binding* (*p*-value = 0.08; MF) (the complete information regarding these terms is available in the Additional file 3).

For the purpose of prognosis, the most interesting terms are evidently those identified as enriched in SCD. Thus, the term *structural constituent of muscle* may have potential for prognosis, given that it occurs more frequently in SCD patients than in no-SCD patients. Nevertheless, the fact that the corrected *p*-value is above the significance level and that the term is not particularly informative in respect to HCM, limits the confidence with which this term can be used for that purpose.

### Study limitations and future work

The results obtained can be explained by the following factors: a) the genetic data can be insufficient to prognosticate the occurrence of SCD; b) the genetic data may not be fully explored; and c) the dataset used may not be the most appropriate to test the methodology. The first two factors are related to the HCM case-study. Firstly, and as already referred, the occurrence of SCD is currently not predictable solely based on genetic data, and thus an enrichment analysis has to be performed considering the clinical data and clinical controlled vocabularies before a final evaluation of the methodology can be made. This analysis will be very important to understand if the inclusion of clinical data is really imperative. Since the HCM dataset is already mapped to clinical vocabularies (NCIt and SNOMED-CT), these vocabularies will be tested next. Secondly, in this initial test we only considered the existence or absence of mutations in the genes, but the type and number of mutations can also be a useful source of information. For example, it is known that some mutations are associated with a benign outcome (i.e. no occurrence of SCD) whereas others with a malignant outcome. It has also been reported that the occurrence of mutations in some genes is associated with a higher incidence of SCD than in others [19]. All of these aspects can be taken into consideration when calculating the frequencies of annotation or even be added as features to the dataset. It is important to notice, however, that we are not concerned with pleiotropic effects. It is known that some HCM mutations have different phenotypic manifestations in different patients, and different manifestations should also be expected if a patient has multiple mutations. Nevertheless, the goal of this analysis is to obtain profiles that provide a global characterization of the patients in respect to an event, and not to perform a precise evaluation of each patient in terms of his mutations. Regarding the genetic enrichment analysis, that global characterization should be in terms of the functions and processes most frequently affected in the event-positive patients. Finally, it cannot be overlooked the possibility that to evaluate the true potential of the prognosis methodology we might need to test it with other datasets. This is due to the reduced number of patients in the dataset tested, in particular of SCD patients.

In respect to the methodology itself, there is also one factor that needs to be taken into account when interpreting the results, that is how the missing values were dealt with. In terms of the genetic features, missing values are mutations associated with HCM that were not tested. Due to the sparseness of the dataset, it was not feasible to simply eliminate the mutations not tested or the patients with mutations not tested. Consequently, we considered these mutations as having a negative value, i.e. that they were not present in the patient. This approach allows us to exploit all the available data and to obtain an informative characterization of the patients. It is important to stress out that an evaluation of all the patients for all the mutations is almost never done. On the one hand, more mutations tested might result in an increase in the number of genes analyzed, possibly leading to an increment in the number of terms tested and, consequently, in the terms found enriched. On the other hand, it might result in an increase in the frequency of annotation of the terms in the study set of the enrichment profiling analysis, and in both the study and the population sets in the differential enrichment analysis. In the profiling analysis, this increase would result in the strengthening of the confidence in the results since we would increase the difference of annotation frequency between the study set and the population set. In the differential analysis, the results might be more strongly altered, since both sets of annotation frequencies would have to be recalculated.

Another relevant factor in the methodology is that a Singular Enrichment Analysis does not take into account the existence of relations between the genes. Since a Modular Enrichment Analysis addresses this issue, we will also test this approach.

By applying a methodology that relies in controlled vocabularies we may have to work with incomplete annotations, as well as with a set of ontology terms that might not provide the level of detail necessary to fully characterize the patients. In respect to the possibility of incomplete annotations, we tried to deal with it by considering all types of annotation, including inferred from electronic annotation, even with the risk of introducing some annotation errors. In respect to the possibility of an insufficient level of detail, it can be overcome by considering more than one vocabulary for the same domain of knowledge, which we will do when analyzing the clinical features.

The enrichment analysis with the clinical data was not yet performed due to the difficulty of defining a population set annotated with terms from clinical vocabularies, as discussed by LePendu *et al.*[6]. In our analysis, this problem presents itself when considering the implementation of the equivalent of the profiling analysis, in which a group of patients would be characterized in terms of their phenotype, based on their clinical information. Considering the approach suggested by the same authors, and that we are currently implementing, the problem can be overcome by exploiting the identification of gene-clinical vocabulary annotations in the PubMed articles that originated the gene-GO annotations. Although the equivalent of the differential enrichment analysis can be more readily implemented, its application with the clinical data presents one of the same limitations found with the genetic data, i.e the existence of a great overlap of annotations between the SCD and no-SCD patients.

## Conclusions

In this article we presented the application of enrichment analysis in a prognosis methodology. The goal of the enrichment analysis was to identify a set of features that might assist in the differentiation of patients for whom a disease-specific event occurred from the patients for whom it did not.

The application of the enrichment analysis was tested with genetic data from patients with the disease hypertrophic cardiomyopathy (HCM), and using the Gene Ontology. The event under analysis was the occurrence of sudden cardiac death (SCD), which is the most severe manifestation of HCM.

The implementation of the analysis was adapted to the fact that we were not studying a single set of genes, but rather several, one from each patient. This adaptation was tested in two enrichment analysis: an enrichment profiling comparing the genes mutated in SCD (or no-SCD) patients with all protein-coding genes in the same group of patients; and a differential enrichment comparing the genes mutated in SCD (or no-SCD) patients with the genes mutated in all HCM patients.

Overall, the results obtained indicate that the enrichment profiling analysis is useful for the characterization of patients, as it allowed the identification of meaningful terms associated with HCM. Notwithstanding, a full evaluation of its potential for prognosis purposes requires that some aspects are taken into consideration. One of such aspects is the fact that in this first implementation, only genetic data was analyzed for enrichment. However, the disease used as case-study cannot be prognosticated solely based on this data, and thus the enrichment analysis has to be performed in both the genetic and the clinical domains. Another aspect is that more information might be extracted from the genetic data in addition to the number of genes mutated, namely the type of mutations (i.e. if they are benign or malignant in respect to the event analyzed) or even the number of mutations per gene. Finally, the methodology itself might need to be tested in other datasets, due to the characteristics of the one used, such as the reduced number of patients and the high number of missing values.

## Methods

### Singular enrichment analysis

The enrichment analysis approach most commonly used is the Singular Enrichment Analysis (SEA). The statistic test underlying this approach is normally the Fisher’s exact test, and the distribution considered when working with small datasets is the hypergeometric distribution. This distribution is applied to situations of sampling without replacement from a finite population when considering that the population elements are in one of two possible states. Translating this to the enrichment analysis, the goal is to evaluate if the genes in the population set are annotated with a term *t*, which means that the two possible states for a gene are: being annotated with the term, and not being annotated with the term. When drawing a sample of genes from the population (thus forming the study set), the objective is then to evaluate if the probability of annotation with term *t* is higher in this sample than would be expected by chance. The expected frequency of annotation is given by the knowledge of the population set, and if the frequency of annotation in the sample is higher than in the population, then term *t* might be used to explain the study set. In this type of analysis, what is being calculated is the probability of observing at least *n* genes in the study set annotated with term *t*, given the knowledge of: the size of the study set, the size of the population set, and the number of genes in the population set annotated with *t*[2]. For a term to be considered enriched in the study set, the *p*-value obtained from the Fisher’s test has to be lower than a significance level, which is normally considered to be 0.05 or 0.1.

The terms tested in this manner are not only those that directly annotate the genes, but also their ancestors. Given the high number of tests that are performed with resources such as the Gene Ontology (with more than 38,000 terms on January, 2013), a multiple-testing correction is necessary to reduce the possibility of false-positive results. The most conservative multiple-testing correction is the Bonferroni correction, which is obtained simply by multiplying the calculated *p*-value by the number of tests performed.

### Enrichment analysis: from genes to patients

The purpose of the methods here described is to analyze patients suffering from a given disease in respect to their predisposition to suffer a disease-related event. The disease and the event are characterizable with clinical and genetic data, and this data is analyzed in terms of ontology terms enrichment. The clinical data includes features such as symptoms and measurements, whereas the genetic data refers to the presence or absence of mutations.

The methods with which we perform the enrichment analysis with the genetic data have been adapted from the standard implementation used in existing enrichment analysis tools. The following two are the main differences between the standard enrichment analysis and the one described here. In the standard analysis: 

•Only one set of genes is analyzed, such as the genome of an organism. In our analysis, several sets of genes are taken into consideration, exactly one for each patient.

•The frequency of annotation of a term is given by the number of genes annotated with that term. In our analysis, the frequency of annotation is given by the number of mutated genes annotated with the term.

#### Enrichment profiling

The purpose of this analysis is to characterize the genotype of a group of patients (e.g. the patients positive for a disease-related event), based on the set of mutations the patients have. Since the knowledge of these mutations is normally not available for the complete genome of a patient but only for a set of genes associated with the disease under analysis, the characterization is performed by comparing the information of the genes mutated in the patients with the complete set of genes in the genome.

Given a group of patients, for each of which is known his/her set of mutations, and the set of Human protein-coding genes, the enrichment profiling analysis is performed as follows: 

1. Define the population set as the union of the genes in the genome of all the patients.

2. Define the study set as the union of the genes mutated in all the patients.

3. Find all GO terms annotating at least one gene mutated in the patients.

4. Calculate the population set frequency of annotation (PFreq) of term *t* as follows:

$$\text{PFreq}(t)=\overset{n}{\underset{1}{\sum }}\text{count}(\text{gene}(t))$$

where *n* is the total number of patients, and *gene(t)* is a gene annotated with *t* (see Figure 2).

5. Calculate the study set frequency of annotation (SFreq) of term *t* as follows:

$$\text{SFreq}(t)=\overset{n}{\underset{1}{\sum }}\text{count}(\text{mut}\_\text{gene}(t))$$

where *mut_gene(t)* is a mutated gene annotated with *t*.

6. Apply Fisher’s exact test to calculate the probability of enrichment of term *t*.

7. Perform a multiple-testing correction (e.g. Bonferroni) over the *p*-values obtained.

8. Consider term *t* as enriched in the study set if *p*-value(*t*) < *α* (e.g. 0.05 or 0.1).

#### Differential enrichment

The purpose of this analysis is to identify differentiating features between a group of patients with a particular characteristic, for example being positive for a disease-related event, and all the patients with the disease. This analysis is also based in the set of mutations the patients have, considering the mutations in the study group vs. the mutations in all the patients.

The implementation of this analysis is very similar to that of the enrichment profiling, with the differences presented below.

Given a group of patients with a disease, a sub-group of those patients with a study characteristic, and the set of mutations in each group: 

1. Define the population set as the union of the genes mutated in the group of patients with the disease.

2. Define the study set as the union of the genes mutated in the sub-group of patients with the study characteristic.

3. Find all GO terms annotating at least one gene mutated in the sub-group of patients.

4. Calculate the population set frequency of annotation (PFreq) of term *t* as follows:

$$\text{PFreq}(t)=\overset{n}{\underset{1}{\sum }}\text{count}(\text{mut}\_\text{gene}(t))$$

where *n* is the total number of patients with the disease, and *mut_gene(t)* is a mutated gene annotated with *t*.

5. Calculate the study set frequency of annotation (SFreq) of term *t* as follows:

$$\text{SFreq}(t)=\overset{n}{\underset{1}{\sum }}\text{count}(\text{mut}\_\text{gene}(t))$$

where *n* is the number of patients in the sub-group with the study characteristic.

6. to 8. Do as in the enrichment profiling.

### Genetic enrichment analysis applied to HCM

### HCM dataset

The HCM dataset is composed by clinical and genetic features characterizing 83 patients, which was previously collected from Portuguese hospitals and molecular biology research laboratories. From these 83 patients, 14 are positive for SCD and the remaining 69 are negative for SCD. Table 7 shows the complete list of clinical features, and Table 8 the percentage of patients with known values for those features. From the total set of clinical features, the following three were used to define which patients are positive for SCD: *sudden death*, *resuscitated sudden death*, and *cardioverter defibrillator*. The first two indicate if the patient suffered a sudden cardiac death, either resuscitated or not, whereas the third indicates if the patient has an implanted cardioverter defibrillator. This device prevents the occurrence of SCD by delivering an electric charge when cardiac arrhythmia is detected, and it is implanted after a resuscitated sudden death occurred or when there is a very high risk of SCD occurrence. Patients are then considered positive for SCD if they are positive for at least one of the three features. Considering the three features instead of just two resulted in an increase of 4 SCD positive patients.

**Table 7:** Features used for the clinical characterization of the HCM patients

**Clinical feature**	**Feature value**	**SCD (%)**	**no-SCD (%)**
**Sudden death (SD)**	True	5 (36)	0
	False	9 (64)	69 (100)
**Resuscitated SD**	True	3 (21)	0
	False	8 (57)	69 (100)
**Cardioverter defibrillator**	True	9 (64)	0
	False	2 (14)	69 (100)
**Non-sudden death**	True	0	0
	False	14 (100)	69 (100)
**Obstructive HCM**	True	4 (29)	8 (12)
	False	1 (7)	17 (25)
**Non-obstructive HCM**	True	1 (7)	17 (25)
	False	4 (29)	8 (12)
**SD family history**	True	3 (21)	1 (1)
	False	2 (14)	25 (36)
**HCM form**	Familial	9 (64)	32 (46)
	Sporadic	2 (14)	37 (54)
**Blood pressure**	Normal	4 (29)	22 (32)
	Hypotension	0	1 (1)
	Hypertension	0	5 (7)
**Gender**	Male	6 (43)	41 (59)
	Female	5 (36)	25 (36)
**Age**	[0,20]	0	5 (7)
	]20,40]	2 (14)	11 (16)
	]40,60]	3 (21)	15 (22)
	> 60	3 (21)	10 (14)

For each feature are indicated its possible values, the number of SCD and no-SCD patients that have them, and the respective percentages. The total number of SCD patients is 14, whereas of no-SCD is 69. (See Table 8 for the percentage of patients with known values for each of the features).

**Table 8:** Percentage of SCD and no-SCD patients that have a known value for each clinical feature

**Clinical feature**	**SCD**	**no-SCD**
**Sudden death (SD)**	100	100
**Resuscitated SD**	79	100
**Cardioverter defibrillator**	79	100
**Non-sudden death**	100	100
**Obstructive HCM**	36	36
**Non-obstructive HCM**	36	36
**SD family history**	36	38
**HCM form**	79	100
**Blood pressure**	29	41
**Gender**	79	96
**Age**	57	61

The genetic features are the mutations associated with the disease, in a total of 569, and are represented as Boolean variables. From this set of mutations, only 78 were found in the HCM patients. These 78 mutations occur in 7 distinct genes (shown in Table 9), all of which are mutated in at least one patient without SCD (no-SCD). In the case of the SCD patients, only 4 of those 7 genes are mutated in at least one of the patients: MYBPC3, MYH7, CSRP3, and TNNT2. The number of mutations identified per patient ranges from 1 to 5, with an average value of 1.8.

**Table 9:** Genes used for the genetic characterization of the HCM patients

**Gene**	**SCD**	**no-SCD**	**GO annotations**
**MYBPC3**	4	25	202
**MYH7**	9	36	192
**CSRP3**	1	4	138
**TNNT2**	2	20	178
**TNNI3**	0	13	173
**MYL2**	0	1	133
**MYH6**	0	1	251

For each gene in indicated the number of SCD and no-SCD patients with at least one mutation in it, as well as the number of Gene Ontology (GO) annotations. The total number of SCD patients is 14, whereas of no-SCD is 69.

The genotyping of the patients was done in two manners: with a microarray able to detect 508 mutations associated with HCM, and a technique called high-resolution melting analysis (HRM) [20] followed by sequencing. The HRM analysis was used to analyze individual exons to indentify the presence of mutations, whereas the sequencing allows the identification of the exact mutation. Some of the patients were analyzed with both techniques, whereas others with only one of the techniques. HRM can be used to test for mutations not present in the microarray and/or to confirm the results obtained with the microarray. One of the reasons to use HRM instead of the microarray is that when patients are tested after a family member was diagnosed, only the mutations found in this one are searched for. Additionaly, the identification of only one mutation is sufficient for a positive diagnosis, and the overall process is cheaper.

### GO annotations

The set of genes in the Human genome was obtained from the GeneCards Database [21], the set of terms from the Gene Ontology [1] and the set of GO annotations from the GOA database [22], as of the releases of October 4th, 2012. From the total set of Human protein-coding genes, only 18,759 were annotated with GO terms. All types of GO annotations were considered, including inferred from electronic annotation.

The enrichment analysis was performed for the three types of GO terms: biological process, molecular function, and cellular component. In order to filter out uninformative GO terms, we considered only terms with information content (IC) above 60%. The IC of a term *t* is given by the expression [23]:

$$\text{IC}(t)=-{\text{log}}_{2}\frac{f(t)}{f(\text{root})}$$

where *f(t)* is the annotation frequency of the term (i.e. the number of distinct gene products it annotates) and *f(root)* is the frequency of annotation of the root term of the GO (which corresponds to the total number of annotated gene products). In this work, we used the annotations to Human genes to compute the IC, including annotations with all evidence codes. In order to obtain a normalized IC, we divided the IC values by the scale maximum (${\text{log}}_{2}f(\text{root})$).

### Enrichment analyses

Four enrichment experiments were performed, two enrichment profiling analysis and two differential enrichment analysis, as presently described in accordance with the steps previously indicated for each analysis. Enrichment profiling for the group of SCD patients: 

1. Population set: 18,759 genes × 14 patients

2. Study set: 16 mutated genes, corresponding to 4 distinct genes (MYBPC3, MYH7, CSRP3, and TNNT2)

3. GO terms obtained for the previous 4 genes

4. PFreq: number of genes annotated with *t* in the 14 patients

5. SFreq: number of mutated genes annotated with *t* in the 14 patients

Enrichment profiling for the group of no-SCD patients: 

1. Population set: 18,759 genes × 69 patients

2. Study set: 100 mutated genes, corresponding to 7 distinct genes (MYBPC3, MYH7, CSRP3, TNNT2, TNNI3, MYL2 and MYH6)

3. GO terms obtained for the previous 7 genes

4. PFreq: number of genes annotated with *t* in the 69 patients

5. SFreq: number of mutated genes annotated with *t* in the 69 patients

Differential enrichment for the HCM patients and the sub-group of SCD patients: 

1. Population set: 116 mutated genes, corresponding to the 7 distinct genes mutated in the 83 patients (MYBPC3, MYH7, CSRP3, TNNT2, TNNI3, MYL2 and MYH6)

2. Study set: 16 mutated genes, corresponding to the 4 distinct genes mutated in the 14 SCD patients (MYBPC3, MYH7, CSRP3, and TNNT2)

3. GO terms obtained for the 7 genes

4. PFreq: number of mutated genes annotated with *t* in the 83 patients

5. SFreq: number of mutated genes annotated with *t* in the 14 SCD patients

Differential enrichment for the HCM patients and the sub-group of no-SCD patients: 

1. Population set: 116 mutated genes, corresponding to the 7 distinct genes mutated in the 83 patients

2. Study set: 100 mutated genes, corresponding to the 7 distinct genes mutated in the 69 no-SCD patients

3. GO terms obtained for the 7 genes

4. PFreq: number of mutated genes annotated with *t* in the 83 patients

5. SFreq: number of mutated genes annotated with *t* in the 69 no-SCD patients

In all the analyses a Bonferroni correction was performed, and 0.1 was the confidence level considered.

## Competing interests

The authors declare that they have no competing interests.

## Authors’ contributions

CMM conceived and implemented the methodology, and drafted the manuscript. ATF and FMC participated in the conception of the methodology, in the draft and revision of the manuscript. All authors read and approved the final manuscript.

## Supplementary Material

Additional file 1 **Full results of the enrichment profiling for SCD patients.** For each term is indicated: GO accession number (GO acc), term name, *p*-value without multiple-testing correction (noCorr), *p*-value with Bonferroni correction, annotation frequency in the study set, annotation frequency in the population set, and the information content (IC). Click here for file

Additional file 2 **Full results of the enrichment profiling for no-SCD patients.** For each term is indicated: GO accession number (GO acc), term name, *p*-value without multiple-testing correction (noCorr), *p*-value with Bonferroni correction, annotation frequency in the study set, annotation frequency in the population set, and the information content (IC). Click here for file

Additional file 3 **Full results of the two differential enrichments: HCM patients and the sub-group of SCD patients; HCM patients and the sub-group of no-SCD patients.** For each term is indicated: GO accession number (GO acc), term name, *p*-value without multiple-testing correction (noCorr), *p*-value with Bonferroni correction, annotation frequency in the study set, annotation frequency in the population set, and the information content (IC). Click here for file
